# Parental Impact on Child Physical Activity and Sedentary Time in Appalachian North Carolina

**DOI:** 10.13023/jah.0203.05

**Published:** 2020-07-19

**Authors:** Allison V. Farrell, Richard W. Christiana, Rebecca A. Battista, J.Joy James

**Affiliations:** Appalachian State University

**Keywords:** Appalachia, North Carolina, sedentary time, children, adolescents, physical activity

## Abstract

**Introduction:**

Physical activity has positive health benefits across the lifespan including reduced rates of chronic disease. Despite having ample availability of outdoor space for physical activity in the Appalachian Mountain region, there are low rates of physical activity along with high rates of sedentary time and increased prevalence of overweight individuals across all age groups. Therefore, there is a need to understand the factors that influence family’s physical activity and sedentary time.

**Purpose:**

To assess whether parental attitudes and behaviors influence children’s physical activity and sedentary time.

**Methods:**

The current study was a secondary analysis of the baseline data from a pilot study of a pediatrician prescription program for outdoor physical activity. Parents (*N* = 70) with children aged 5–13 years living in a county served by a single-pediatrician office completed surveys in the pediatrician’s office during a well-child visit. The survey included questions related to parental attitudes toward children’s physical activity and the physical activity and sedentary time performed by the parent and their child.

**Results:**

Parent sedentary time was the only factor that had an impact on child sedentary time, with 18% of the variance in children’s sedentary time being explained by parent sedentary time. No factors predicted children’s physical activity.

**Implications:**

To decrease child sedentary time, interventions should focus on reducing parental and joint parent–child sedentary time.

## INTRODUCTION

Physical activity is an important component of health as it reduces the risk of various chronic diseases both physical and mental.^1^ Despite the health benefits only 24% of children aged 6 to 17 years in the U.S. meet the recommended 60 minutes of daily moderate to vigorous physical activity.^2^ As a result, determining ways to increase physical activity and understanding the influences on children and adolescent physical activity behavior has seen much attention.

The family unit has been a recent area of interest as it may impact children’s physical activity, particularly the parent’s role on their child’s physical activity.^3^,^4^ For example, Trost et al. discovered parental physical activity, enjoyment in participation in physical activity, and physical activity performance were positively associated with parental support for child physical activity. However, Bauer et al. suggested that parents modeling physical activity does not necessarily mean their child will also be physically active.^3^,^4^ Nonetheless, it has been suggested that parental support, particularly tangible (e.g., transportation, equipment) and intangible (e.g., verbal support, encouragement) social support is positively associated with child physical activity.^3^–^5^

Along with physical activity, sedentary time may also impact health outcomes. Similar to the time spent being active, the time spent being sedentary (e.g., sitting at work or school, watching television, or playing video games) may also affect children’s health. Sedentary time poses great health risks as children develop to adulthood, including low metabolism and poor musculoskeletal health and is associated with all-cause mortality and cardiovascular disease, independent of physical activity.^6^,^7^ Sedentary time is defined as “the time spent for any duration (e.g., minutes per day) or in any context (e.g., at school or work) in sedentary behaviors.”^8^ Increased time spent in sedentary activities is predominant in both adults and children with the concern that a decrease in physical activity may result in engaging in more sedentary time.^6^ If the focus is on the family unit, perhaps parental sedentary time may actually influence the child.

Bauer et al.^4^ suggested that children tend to mirror their parents in sedentary time, although children may not mirror their parents in physical activity. Though parental role-modeling of physical activity may have little effect on children, parental beliefs about physical activity have been shown to influence children’s physical activity and sedentary time regardless of children’s perception of these beliefs.^3^–^5^,^9^ Additionally, it has been suggested parents may also want to consider placing limits on sedentary activities, which may decrease child sedentary time.^10^

Thus, the parents physical activity and sedentary time and their current beliefs or boundaries regarding these activities may influence their child’s time spent in either physical activity or sedentary activity.

However, there is limited research investigating the relationship between parent physical activity on child physical activity; parent sedentary time on child sedentary time; and parent limiting sedentary time on child’s physical activity and sedentary time. This lack of research is especially pronounced for rural Appalachian populations that have been found to have youth with high rates of poor health behaviors.^11^,^12^ Therefore, this study explored whether children’s physical activity and sedentary time were influenced by parental physical activity, sedentary time, parental beliefs about the importance of children’s physical activity, and parental limits on children’s screen time in rural Appalachia?

## METHODS

### Study Design and Population

This cross-sectional study is a secondary data analysis of baseline data from a pilot study of a pediatrician prescription program for outdoor physical activity for children. Full study procedures are described elsewhere.^13^ Parents with children aged 5 to 13 years were recruited from the single pediatrician’s office in a county in the Appalachian Mountains in western North Carolina during a well-child visit. Parents completed a baseline survey asking for informed consent prior to seeing the pediatrician. All study procedures were approved by the Institutional Review Board at Appalachian State University. A total of 70 parents were recruited.

### Measures

The survey collected data on the age of the child, parent race/ethnicity, total household income, and highest level of education in the home. Survey questions assessed child and parent physical activity, sedentary time, whether parents regularly limit their child’s screen time (watching TV, playing video games, using the computer), and parental beliefs about children’s physical activity (Table 1).

Parent and child moderate-to-vigorous physical activity (MVPA) was assessed using two questions from the Godin Leisure-Time Exercise Questionnaire (LTEQ) that asked about weekly time spent in moderate and vigorous physical activities.^14^ Two variables were created for child and parent MVPA by multiplying the time spent in vigorous physical activity by two and then summing with the time spent in moderate physical activity. Child’s sedentary time was assessed using two questions that asked the amount of time during an average weekday and weekend day the child spends in sedentary behaviors. Parent sedentary time was assessed using a similar question adapted for adults. Parent and child’s sedentary time per week was estimated by the sum of weekday sedentary time multiplied by five and weekend day sedentary time multiplied by two. Parental limits placed on children’s screen time was assessed by three questions. To allow for regression analysis, each of these questions was converted to a dichotomous variable where “0” was “never to sometimes” and “1” was “very often to always.”

Items assessing sedentary time and parental limits placed on children’s screen time were adapted and tested for comprehension with a separate small group of parents. Parental beliefs related to the importance of children’s physical activity was measured using the Parental Beliefs about Physical Activity scale developed by Lee et al.^15^ To allow for regression analysis, each of these instrument items was converted to a dichotomous variable where “0” was “strongly disagree to neither disagree nor agree” and “1” was “agree to strongly agree.”

### Data Analysis

Multiple linear regression (*p*<0.05) was conducted to assess whether parent physical activity, sedentary time, beliefs about child’s physical activity, and limiting child’s screen time predicted child’s physical activity and sedentary time. All statistical analyses were conducted using IBM SPSS Statistics, version 24. A hierarchical multiple regression was then conducted on models that were statistically significant to assess whether significance was maintained with the inclusion of parent race/ethnicity, the highest level of education in the home, total household income, and child’s age as control variables.

## RESULTS

Despite being in a small town in the Appalachian region of western North Carolina, the participants were mostly white and highly educated, with more than half of parents having a college degree or higher, (Table 2). Most households consisted of married parents, with a higher socioeconomic status and a household income of $55,000 per year. This is not a typical representation of the county as a whole based on census data, with residents outside of the town having lower levels of education and household income.^16^ However, the race and ethnicity of the participants is representative of the area, which is predominantly white.

The bivariate associations reveal strong relationships between child’s sedentary time and parent sedentary time, between the three variables assessing parents limiting child’s time watching TV, playing video games, and using the computer as well as several of the parental belief variables (i.e., *r* > Cohen’s standard for a large effect size, *r* > 0.371).^17^ The associations between child’s sedentary time and one of the parental belief variables as well as parents limiting child’s time watching TV were smaller (*r* = − 0.253 and − 0.250, respectively) and represent a medium effect size (i.e., the standard for a medium effect size *r* = 0.243). The associations between parent physical activity and parent sedentary time (*r* = −0.268) and between two of the parental belief variables (*r* = 0.284) represent a medium effect size.

A multiple linear regression to predict child’s physical activity based on parent physical activity, sedentary time, beliefs about child’s physical activity, and limiting child’s screen time was found not significant (*F* (11,56) = 1.04, *p* = 0.43).

A second multiple linear regression to predict child’s sedentary time based on parent physical activity, sedentary time, beliefs about child’s physical activity, and limiting child’s screen time was significant (*F* (11,55) = 3.71, *p* < 0.00), with an *R*^2^ of 0.43. More specifically, parent sedentary time, one parental belief variable (i.e., parents responsibility to help their child find physical activity), limiting child’s time watching TV, and limiting child’s playing video games made statistically significant, unique contributions to the estimation of child’s sedentary time (*p*<0.05) with parent sedentary time positively associated with child’s sedentary time and the one parental belief variable, limiting child’s time watching TV, and limiting child’s time playing video games negatively associated with child’s sedentary time. The relative contribution of the independent variables was evaluated through the interpretation of squared semi-partial coefficients.^18^,^19^ Results indicated parent sedentary time made the largest unique contribution and revealed a predictive efficacy of over 1.5 times larger of the one parental belief (0.116/0.070 = 1.7), and two times larger than that for limiting child’s time watching TV (0.116/0.049=2.4) and playing video games (0.116/0.050=2.3).

Effect sizes were calculated for the four predictors using Cohen’s *f* ^2^, where values of 0.02 represent a small effect, values of 0.15 equal a medium effect, and values of 0.35 denote a large effect.^17^ Results illustrate parent sedentary time had a medium-to-large effect size (*f* ^2^ = 0.20), the one significant parental belief had a small-to-medium effect size (*f* ^2^ = 0.12), and both limiting child’s time watching TV and playing video games had small effect sizes (*f* ^2^ = 0.09) in predicting child’s sedentary time.

A hierarchical multiple regression stratified by child’s age to predict child’s sedentary time based on parent sedentary time was calculated due to differences in parental influence among children and adolescents. Child’s age was stratified into two age groups, those aged 5–9 years and those aged 10–13 years, based on the literature indicating that sedentary time tends to increase at early adolescence.^20^ Parent race/ethnicity, highest level of education, and total household income were controlled for in the regression analysis using a two-stage model recommended by Keith.^21^ Parent sedentary time, the belief that it is the parents responsibility to help their child find physical activity, and parent’s role in limiting child’s TV and video game use were entered in the second stage. Distributional statistics are presented in Table 3 for the predictors and criterion. Results from the hierarchical multiple regression indicate a statistically significant increase in the variance explained in child’s sedentary time among the group aged 10–13 years, *Δ*^2^=.35,*F* (4,23) = 4.06, *p* < 0.01, but not the 5–9 year old group, *F* (4,25) = 0.88, *p* = 0.49 (Table 4). In fact, only parent sedentary time was positively associated and made a significant unique contribution to the prediction of child’s sedentary time among the group aged 10–13 years (*p* <0.01) with a large effect size (*f* ^2^ =0.44).

## IMPLICATIONS

This study investigated the impact of parent physical activity, sedentary time, parental beliefs about children’s physical activity, and parents limiting child’s sedentary activities on child physical activity and sedentary time. Parent sedentary time was the only significant predictor of child sedentary time, but only among early adolescents (aged 10–13 years). No significant predictors of child sedentary time were found among children (aged 5–9 years). Parent physical activity, sedentary time, beliefs, and limits on child’s sedentary activities did not predict child physical activity.

Parental physical activity and its influence on child physical activity is varied, with conflicting findings. The results that parental physical activity is not related to child physical activity, is consistent with a majority of current literature. Parental modeling of physical activity, simply parents being physically active, has been found to not be related to their child’s physical activity.^3^,^4^,^9^,^22^–^24^ However, several studies have shown a positive relationship.^25^–^27^ For example, Zarychta et al. concluded parents that modeled a healthy diet and physical activity were more likely to have adolescents who were more physically active and had overall healthier lifestyles.^25^ Similarly, Fuemmeler et al. found that children with two physically active parents had significantly higher MVPA than children with two inactive parents,^26^ while other researchers determined that physical activity performed as a family may relate to increased child physical activity.^5^,^28^ Thus, the relationship between parent and child physical activity may be more complex than simple parent modeling of behavior.

In terms of sedentary time, the current study found that parents’ sedentary time was a significant predictor of children’s sedentary time among early adolescents, but not among children. In fact, the effect size of parents’ sedentary time among early adolescents was large (*f*^2^ = .54). These results are similar to Jago et al. when looking at both physical activity and sedentary time.^22^ However, Jago et al. determined, television viewing was the only factor associated between parents and children.^22^ Additionally, Dunton et al. found that parents and children are more likely to engage in sedentary time together (92.9 minutes/day) than physical activity (2.4 minutes/day).^28^

When examining parental physical activity, beliefs about their child’s physical activity on adolescents’ physical activity and sedentary time, Bauer et al. concluded there is a complicated relationship between parent and child sedentary time, with parental encouragement and perception not having an impact on child sedentary time.^4^ In other words, parents may have a greater impact on adolescent sedentary time compared to child sedentary time because of decreasing physical activity levels as children age.^4^ Nonetheless, the evidence regarding the influence of parental beliefs about children’s physical activity on behavior is conflicting. For instance, Kimieck et al. identified children’s perceptions of their parents’ beliefs were not related to their own physical activity and suggested parental support, specifically belief in their child’s physical activity competency, was related to increased physical activity among children.^9^ Yet, others revealed parental encouragement was the main factor impacting child physical activity.^4^,^29^ Thus, there may be a complicated relationship between parental beliefs and child physical activity and parents must take an active role in their child’s physical activity through supporting and motivating physical activity behavior.

Most studies have examined only physical activity,^3^,^9^,^22^,^27^,^30^ with some looking at both physical activity and sedentary time.^4^,^5^,^22^ Few studies have investigated the influence of parental physical activity, sedentary time, beliefs about physical activity, and limiting children’s screen time on children’s physical activity and sedentary time. There is support for the interrelatedness of all of these factors, however the current study is the first to examine all of these factors together.

These results provide evidence for the development of effective interventions to improve the health of youth. Interventions may be designed for the entire family to participate versus only children or only parents. Setting limits on total sedentary time, encouraging physical activity participation, developing these limits with the child’s age in mind and, educating parents about their own sedentary time and its influence on their children’s sedentary time may be worth implementing. Nonetheless, it is important that more children meet physical activity recommendations and less exceed the suggested amount of daily screen time to prevent future health problems. Decreasing parent sedentary time may be valuable in improving the health outcomes of their children. Healthy People 2020, which sets 10-year national objectives for public health in the United States, aims to increase child physical activity and decrease the proportion of children who exceed recommended limits for screen time.^31^ The results from this study can be used by health professionals to help accomplish the objectives of Healthy People 2020.

Overall, this study fills an important gap in the literature regarding the relationship between parental behavior, beliefs, and limits placed on their child’s screen time and child physical activity and sedentary time. Currently, there is a lack of research exploring parent and child sedentary time, with only some focusing on physical inactivity (i.e., not getting an adequate amount of MVPA), as a proxy for sedentary time.^5^,^32^,^33^ It is important that physical activity and sedentary time are observed as separate variables as they are not mere opposites but rather separate factors associated with different health outcomes.^6^,^34^ As such, future research and interventions should approach sedentary time and physical activity separately because they are distinct behaviors.

Specific to Appalachia, there is limited research on parent impact on child physical activity and sedentary time. The Appalachian region is unique in the challenges it faces including being rural and geographically isolated while having low average income and poor health outcomes. Other challenges include limited access to recreation, sports programs, and transportation.^35^ In North Carolina, the Appalachian region has higher levels of physical inactivity and almost half of adults fail to meet physical activity recommendations.^32^,^33^ The results of this study help fill a gap in knowledge on the specific impact of parents on children’s physical activity and sedentary time in Appalachia. This information can be used by health professionals to create programming targeted at improving children’s healthy behaviors that incorporates parents. Future interventions should focus on creating more opportunities for sports, adding recreational programming opportunities for whole families in communities, and providing transportation.

To the best of our knowledge, this study is one of the few that has measured and examined predictors of children’s physical activity and sedentary time separately rather than using physical inactivity as a proxy for sedentary time. However, this study had several limitations. Given the small sample size, conclusions drawn from this data are limited. In addition, subjects came from a single pediatrician’s office, which both limited the sample size and the diversity of subjects. However, the area where this study was conducted only had one pediatric office which serves a three-county area. Another limitation was that parent physical activity and sedentary time were self-reported, while parents reported their child’s physical activity and sedentary time. Parents have been found to often overestimate their child’s physical activity and this could have impacted the results.^36^

**SUMMARY BOX**

**What is already known about this topic?** Although ample research has shown that parents influence children’s health behaviors, there is limited research investigating the relationships between parent physical activity, sedentary time, and limiting children’s sedentary time on children’s physical activity and sedentary time in rural Appalachian populations.

**What is added by this report?** This study fills a gap in knowledge on the impact of parents on children’s physical activity and sedentary time in rural Appalachia. While the results indicated that parent physical activity did not have an impact on child physical activity, parental sedentary time may be one of the many factors that contribute to child sedentary time. Parent sedentary time may have more of an impact on child behavior than physical activity because parents are more often engaged in joint sedentary time than joint physical activity.

**What are the implications for future research?** Future interventions should incorporate decreasing parent sedentary time as a means to lower child sedentary time and include joint parent and child physical activity to increase child physical activity by creating opportunities for sports and recreational programming for whole families.

## Figures and Tables

**Table 1:** Survey Instrument Items

Child Items
About how many hours a day on (school days/weekends or non-school days) does your child spend in sedentary activity (sitting while listening to music, watching TV, playing video games, using a computer or tablet/iPad, doing homework, reading, etc.)?*
How often do you put limits on how much time your child may watch TV?**
How often do you put limits on how much time your child may play video games?**
How often do you put limits on how much time your child may use the computer?**
Kids who do regular physical activity have more self-confidence.***
Kids who do regular physical activity are healthy.***
Kids who do regular physical activity will be healthier adults.***
It is the parents’ responsibility to help their child find physical activity.***
All kids should be physically active every day.***
Parents play an important role in whether their kids are physically active when they grow up.***
**Parent Items**
About how many hours a day do you spend in sedentary activity (sitting while listening to music, reading, watching TV, playing video games, using a computer or tablet/iPad, doing paperwork, driving, etc.)?*

* none, less than 1 hour a day, 1 hour a day, 2 hours a day, 3 hours a day, 4 hours a day, 5 or more hours a day

** never, rarely, sometimes, very often, always

*** strongly disagree, disagree, neither disagree nor agree, agree, strongly agree

**Table 2:** Demographic Characteristics for Parents and Children

Variable	n (%)
**Child**	
Gender	
Female	31(44.3)
Male	39(55.7)
Race/ethnicity	
White	65(92.9)
Hispanic or Latino	3(4.3)
Other*	2(2.9)
**Parent**	
Race/ethnicity	
White	66(94.3)
Hispanic or Latino	1(1.4)
Other*	3(4.3)
Highest level of education of all adults in household	
High school grad or GED	7(10.0)
Some college	10(14.3)
College grad	33(47.1)
Graduate/professional school	20(28.6)
Total household annual income	
Under $15,000	5(7.1)
$15,000–$34,999	9(12.9)
$35,000–$54,999	13(18.6)
Over $55,000	40(57.1)

**Table 3:** Distributional Statistics for Child’s Sedentary Time and Predictors for Hierarchical Multiple Regression

Variable	Group Aged 5–9 Years M(SD)	Group Aged 10–13 Years M(SD)
Child’s Sedentary Time	15.33(4.60)	21.45(8.14)
Parent Race/Ethnicity	1.39(1.41)	1.10(0.54)
Highest Level of Education in Home	5.58(1.32)	6.13(0.85)
Total Household Income	3.64(1.69)	4.19(1.42)
Parent’s Sedentary Time	17.18(9.60)	18.06(10.50)
Parental Belief		
It is the parents’ responsibility to help their child find physical activity	0.94(0.24)	0.90(0.30)
Frequency of Limiting Screen Time		
Watching TV	0 .58(0.50)	0.45(0.51)
Playing Video Games	0.61(0.50)	0.58(0.50)

Note: M = mean, SD = standard deviation, N_5–9yrs_ = 33; N_10–13yrs_ = 31.

**Table 4:** Hierarchical Multiple Regression Analysis Summary for Variables Predicting Child’s Sedentary Time

Variable	*B*	SE *B*	β	*pr* ^*2*^	*f* ^*2*^
**Group aged 5–9 Years**					
**Step 1**					
Parent Race/Ethnicity	0.417	0.561	0.128	0.019	0.073
Highest Level of Education in Home	−0.463	0.757	−0.133	0.013	0.050
Total Household Income	−0.731	0.590	−0.269	0.050	0.194
**Step 2**					
Parent Sedentary Time	0.078	0.086	0.164	0.032	0.126
Parental Belief	−3.08	3.51	−0.162	0.030	0.116
Limiting TV	−6.87	5.09	−0.749	0.068	0.264
Limiting Video Games	6.01	5.04	0.648	0.054	0.209
Constant	18.44	5.12			
**Group Aged 10–13 Years**					
**Step 1**					
Parent Race/Ethnicity	5.13	2.74	0.339	0.115	0.225
Highest Level of Education in Home	−0.113	1.97	−0.012	0.0001	0.0002
Total Household Income	−1.80	1.18	−0.315	0.080	0.156
**Step 2**					
Parent Sedentary Time	0.405	0.134	0.522**	0.285	0.558
Parental Belief	−5.50	5.20	−0.203	0.046	0.090
Limiting TV	−6.55	3.78	−0.407	0.116	0.226
Limiting Video Games	3.71	3.87	0.228	0.038	0.075
Constant	29.64	10.60			

*Note*: *R*^2^_5–9yrs_ = 0.258 (*N* = 33, *p* = 0.49); *R*^2^_10–13yrs_ = 0.511 (*N* = 31, *p* < 0.01), *pr*^2^ = squared semi-partial coefficient, *f*^2^ = Cohen’s (1988) effect size statistic for multiple regression analyses.

* *p* ≤ 0.05,

** *p* ≤ 0.01,

*** *p* ≤ 0.001
